# tRNA evolution from the proto-tRNA minihelix world

**DOI:** 10.1080/21541264.2016.1235527

**Published:** 2016-09-16

**Authors:** Robert Root-Bernstein, Yunsoo Kim, Adithya Sanjay, Zachary F. Burton

**Affiliations:** a Department of Physiology, Michigan State University, E. Lansing, MI, USA; b Troy High School, Troy, MI, USA; c Department of Biochemistry and Molecular Biology, Michigan State University, E. Lansing, MI, USA

**Keywords:** anticodon loop, acceptor stems, D loop, proto-tRNA minihelices, ribosome evolution, rRNA evolution, tRNA evolution, tRNA structure, tRNA microhelices, T loop, V loop

## Abstract

Multiple models have been advanced for the evolution of cloverleaf tRNA. Here, the conserved archaeal tRNA core (75-nt) is posited to have evolved from ligation of three proto-tRNA minihelices (31-nt) and two-symmetrical 9-nt deletions within joined acceptor stems (93 – 18 = 75-nt). The primary evidence for this conclusion is that the 5-nt stem 7-nt anticodon loop and the 5-nt stem 7-nt T loop are structurally homologous and related by coding sequence. We posit that the D loop was generated from a third minihelix (31-nt) in which the stem and loop became rearranged after 9-nt acceptor stem deletions and cloverleaf folding. The most 3´-5-nt segment of the D loop and the 5-nt V loop are apparent remnants of the joined acceptor stems (14 – 9 = 5-nt). Before refolding in the tRNA cloverleaf, we posit that the 3′-5-nt segment of the D loop and the 5-nt V loop were paired, and, in the tRNA cloverleaf, frequent pairing of positions 29 (D loop) and 47 (V loop) remains (numbered on a 75-nt tRNA cloverleaf core). Amazingly, after >3.5 billion years of evolutionary pressure on the tRNA cloverleaf structure, a model can be constructed that convincingly describes the genesis of 75/75-nt conserved archaeal tRNA core positions. Judging from the tRNA structure, cloverleaf tRNA appears to represent at least a second-generation scheme (and possibly a third-generation scheme) that replaced a robust 31-nt minihelix protein-coding system, evidence for which is preserved in the cloverleaf structure. Understanding tRNA evolution provides insights into ribosome and rRNA evolution.

## Introduction

Many defining events in ancient molecular evolution built complex systems from basic, modular subunits leaving a “molecular paleontology” still discernable in current protein and nucleic acid structures and sequences, some 4 billion years after the emergence of life.^1,2^ Starkly simple models have been demonstrated for the ancient evolution of metabolism (TIM barrels (β−α)_8_ and Rossmann fold (β−α)_8_ linear sheets),^3^ multi-subunit RNA polymerases (2-double-Ψ-β-barrel ((ββαβ)_2_ barrel) type),^3-9^, and general transcription factors.^3,6,10^ The observation, for instance, that bacterial σ factors (4-helix-turn-helix (HTH)) are homologs of archaeal/eukaryotic TFB/TFIIB (2-HTH (corresponding to σ HTH_3_-HTH_4_)) suggests a simple model describing evolution of promoter DNA sequences (archaeal/eukaryotic BREs and TATA boxes; bacterial −35 and −10 regions).^3^ Insulin, the insulin receptor and glucose transporters are all constructed from a common set of more primitive glucose-binding modules.^11-13^ A concatenation of tRNA-like modules may form the structure of rRNAs^14,15^ and may form the heart of the peptidyl transferase center (PTC).^16,17^

Here, highly conserved tRNA structures and sequences are analyzed to gain insight into the origins of tRNAs and the dawn of protein synthesis and ribosome-dependent translation. The ribosome can be considered to be a: (1) scaffold, (2) reading head, and (3) translocation apparatus, to accurately and efficiently translate mRNA sequence into protein sequence. Although the prokaryotic ribosome includes 23S, 16S, and 5S rRNA and multiple protein subunits, in terms of its catalytic function in peptide bond formation, the ribosome is considered to be primarily a ribozyme.^18^ The peptidyl transferase activity does not directly utilize any ribosomal proteins and is purely a function of domain 5 (V) of the 23S rRNA. Furthermore, tRNA fulfills a core function in both coding and peptidyl transfer. The growing polypeptide chain is held covalently by an amino acyl linkage to the tRNA in the ribosome P site. Within the PTC, a covalently amino acylated tRNA in the ribosome A site is brought into close proximity to the C-terminal end of the growing polypeptide chain, where the incoming amino acid is covalently joined. Amino acid transfer leaves the tRNA-polypeptide in the ribosome A site and an empty tRNA in the P site. After translocation of the A site tRNA-polypeptide to the P site and, with entry of the incoming mRNA-encoded tRNA-aa to the A site, the next peptide bond can be formed. Because the ribosome and its accessory factors constitute such an intricate apparatus, a reductionist model for evolution of peptide bond formation and complex cellular translation systems may seem inconceivable, yet a simple and step-wise evolutionary process must be assumed.

To simplify the problem for ribosome evolution, therefore, consider a tRNA centric model.^19,20^ According to such a view, because tRNAs covalently attach the elongating polypeptide chain and the incoming amino acid, and because bringing an activated amino acid in proximity to a restrained polypeptide chain is a key step in catalysis, tRNAs might be considered to be a central feature of peptidyl transfer. Here, we (along with others) support this more central role for tRNAs. The PTC of the ribosome has been considered to have pseudo symmetry and may have evolved from ligation of 2-proto-tRNA “minihelices” or “stem-loop-stems;”^16^ also, evolutionary sequence analysis of proto-tRNA appears to support the minihelix model.^17^ 23S, 16S, and 5S rRNA sequences bear many striking similarities to tRNA sequences, indicating that, as expected, rRNA and tRNA are co-evolved.^14,15^

## Results

**Figure 1.**
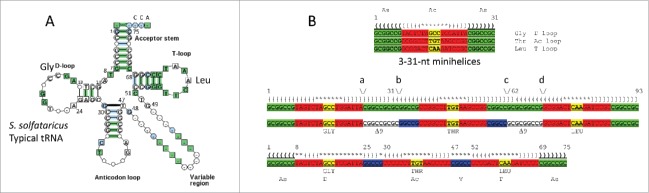
A model for tRNA evolution. (A) A *Sulfolobus solfataricus* typical tRNA structure (similar to a consensus sequence).^25^ The numbering system is based on a 75-nt tRNA core. (B) The model. 3-31-nt minihelices are ligated. A symmetrical 9-nt deletion occurs within two-ligated acceptor stems. Green: 7-nt acceptor stems; blue: 5-nt relics of acceptor stems after deletions; red + yellow: 17-nt microhelices; yellow: anticodons; no highlight: 9-nt symmetrical deletions.

We posit that tRNA evolved from ligation of 3–31-nt proto-tRNA minihelices and two-symmetrical 9-nt deletions where the minihelices were joined (for alternate views see^21-24^) (Fig. 1). In Figure 1A, a “typical” tRNA cloverleaf diagram^25^ is shown for *Sulfolobus solfataricus* (an archaea), numbered according to our model. In Figure 1B, the model is shown to generate a 75-nt tRNA core via ligation of 3-31-nt minihelices and 2-symmetrical 9-nt deletions within ligated acceptor stems (Fig. 1B; green segments). Deletions are posited to occur between positions a and b and c and d (Fig. 1B). In this paper, 17-nt stems (2 × 5-nt) and loops (7-nt) are referred to as “microhelices” (Fig. 1B; red and yellow segments). A 31-nt minihelix is a 17-nt microhelix attached to a 14-nt (2 × 7-nt) acceptor stem. The anticodon (Ac) loop microhelix (30–46) resembles the T loop microhelix (52–68) in sequence and secondary structure (Fig. 1A). We posit that the D loop arose from a refolded 17-nt microhelix (8–24) and a 5-nt remnant of an acceptor stem (25–29). The simple V loop, without insertions or deletions, is 5-nt (47–51). The 5-nt V loop is posited to be a relic of an acceptor stem, which, when folded as a minihelix, paired with the 3´-5-nt segment of the D loop (25–29). According to the model, therefore, the tRNA core is comprised of 2 × 7-nt acceptor stems, 2 × 5-nt acceptor stem relics, and 3–17-nt microhelices. In many archaea and eukaryotes, 3´-CCA is attached enzymatically, so CCA is not considered in the model.

**Figure 2.**
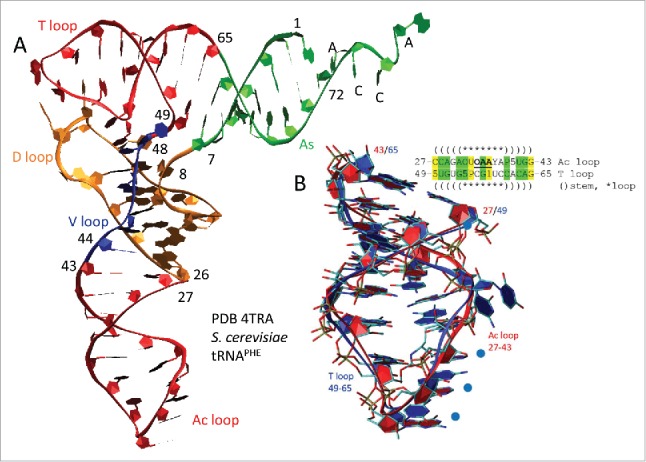
The T loop and the Ac loop are homologs. (A) The tRNA cloverleaf includes two related microhelices: the T loop and Ac loop (red). (B) An overlay of the 17-nt anticodon loop (7-nt) and stem (2 × 5-nt) (red) and the 17-nt T loop (7-nt) and stem (2 × 5-nt) (blue) shows remarkable structural similarity. Because of a 3-nt deletion in the D loop, numbering from within the D loop for *S. cerevisiae* tRNA^PHE^ is reduced by 3-nt compared to the model for 75-nt tRNA evolution (see Fig. 1).^32^ In the sequence, yellow shading indicates DNA-coding identity and green shading similarity. The anticodon is bold and underlined. Five indicates 5-methyl-cytosine. P is pseudo-uridine. One and Y are adenosine derivatives. O is a uracil or a guanosine derivative. Blue circles in (B) indicate anticodon positions.

Because the Ac and T loops resemble one another in sequence and secondary structure (Fig. 1A), a structural comparison of the Ac and T loops was done (Fig. 2). Figure 2A indicates that the Ac and T loops are structurally similar (compare (red) 17-nt microhelices). Figure 2B shows an overlay of the Ac and T loop 17-nt microhelices, which align within 1.9 Å RMSD (root-mean-square deviation) for backbone atoms. We conclude that the Ac and T loops, which are similar in sequence, are close structural homologs.

**Figure 3.**
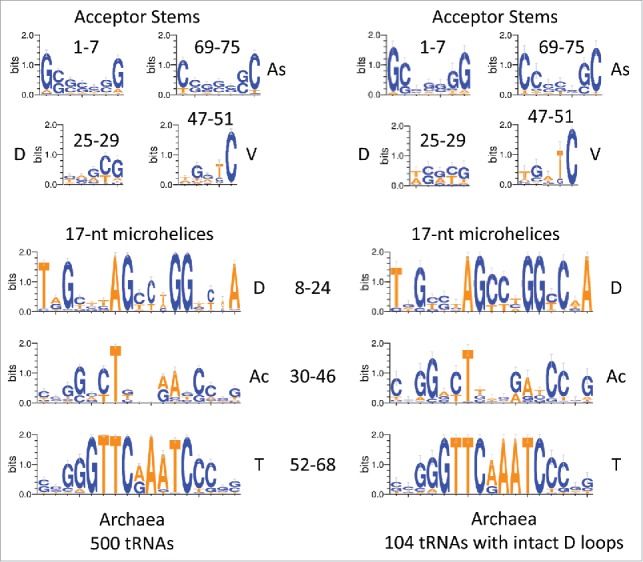
A logo comparison of archaeal tRNAs is shown. The left panel represents 500 archaeal tRNAs. The right panel represents 104/500 total archaeal tRNAs selected for having no deletions in the D loop (104 tRNAs). The numbering is based on the 75-nt tRNA model (Fig. 1).

**Figure 4.**
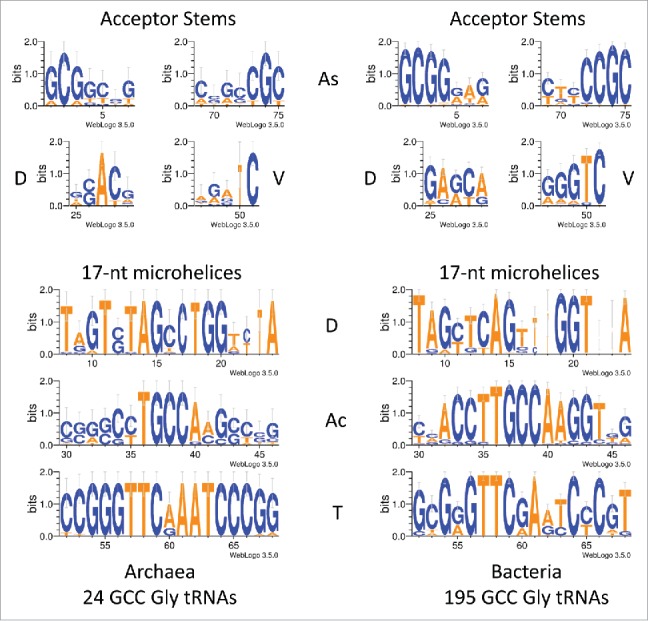
Logo comparisons of archaeal and bacterial tRNA^GLY^ with GCC anticodons.

**Figure 5.**
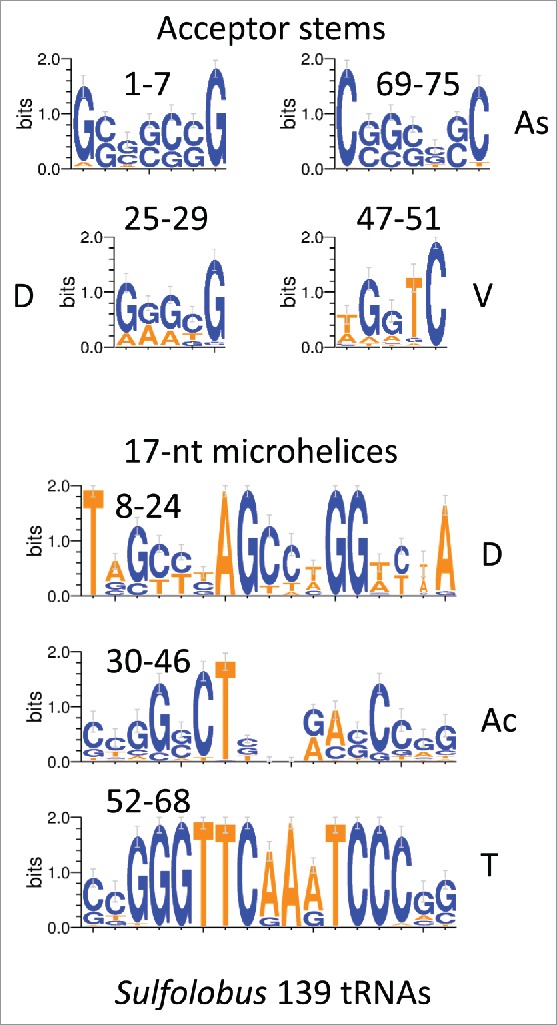
Logo comparisons of archaeal *Sulfolobus* tRNAs (139 tRNAs from three *Sulfolobus* species).

For deeper analysis, archaeal tRNA sequences were collected from the tRNA database,^25^ and logo analyses were done (Figs. 3–5). In Figure 3, archaeal tRNAs (500 tRNAs) and archaeal tRNAs with an intact D loop (104 tRNAs) were collected for logo presentation. Archaea is an ancient domain and compared to bacteria generally shows higher consistency in tRNA sequence. Archaeal tRNAs, therefore, may have preserved more sequence matches to LUCA (the last universal cellular common ancestor) tRNAs. ∼20% of archaeal tRNAs include what we identify as an intact D loop (17-nt microhelix + 5-nt acceptor stem remnant; 8–29) (i.e., 104/500). As we show below, the D loop was also likely derived from a 31-nt minihelix, so we posit that 3–31-nt minihelices were initially ligated in formation of the tRNA core (Fig. 1B).

In Figures 3–5, according to the model (Fig. 1), the 75-nt archaeal tRNA core is broken into 2–7-nt acceptor stems (1–7 and 69–75), 2–5-nt acceptor stem remnants (25–29 (part of the D loop) and 47–51 (V loop)) and 3–17-nt microhelices (8–24 (part of the D loop), 30–46 (Ac loop), and 52–68 (T loop)) (75-nt total). At its 3′ end, the D loop includes a 5-nt remnant of a 7-nt acceptor stem (25–29 relate to 3–7 to which they are aligned in Figs. 3–5). The V loop includes a 5-nt remnant of a 7-nt acceptor stem (47–51 relate to 69–73, to which they are aligned in Figs. 3–5). We posit that, when the Ac loop was a minihelix (i.e., before LUCA), D loop 25–29 paired with V loop 47–51. After D loop refolding in the cloverleaf, of course, there is likely negative evolutionary pressure on D loop pairing to the V loop because D to V loop pairing interferes with D loop folding to form the cloverleaf. Negative selection against D to V loop pairing may partly explain frequent deletions in the D loop and/or insertions (or 1-nt deletions) in the V loop. Acceptor stems are G/C rich, and most adenine substitutions in the D and V loops would also be expected to disrupt pairing (Figs. 3–5). We further posit that, as noted above, the remainder of the tRNA cloverleaf core is broken into 3–17-nt microhelices (positions 8–24 of the D loop, positions 30–46 of the Ac loop, and positions 52–68 of the T loop). Logos are shown for 500 archaeal tRNAs and 104/500 tRNAs selected for having intact D loops. The logos are very similar for both sets of tRNAs, demonstrating the very high conservation of archaeal tRNAs. Because (for the left panel) these tRNAs represent all 20 amino acids, and because each tRNA anticodon represents its own separate lineage, this conservation is remarkable.

Figure 4 shows glycine GCC anticodon tRNA^GLY^ logos in archaea and bacteria. Note that, from LUCA, each tRNA lineage (based on anticodon) is expected to be largely independent from other tRNA lineages, so, as expected, a stronger sequence consensus print is expected for a tRNA^GLY^ GCC logo (Fig. 4) compared to total archaeal tRNA sequences (Fig. 3). The consensus is also strong for a tRNA^GLY^ GCC logo using bacterial tRNA^GLY^ GCC (Fig. 4; right panel). Consistent with our hypothesis, tRNA^GLY^ fits the 75-nt model that we propose for the tRNA core, so the multiple sequence alignment numbering in Figure 4 did not require adjustment to the model, as it did in Figures 3 and 5. Figure 5 shows a logo comparison for 139 archaeal *Sulfolobus* tRNAs from three-species. Because each tRNA anticodon is expected to represent a separate lineage from LUCA, this is a broad alignment. The D and V loops have significant capacity to pair (4/5 positions). Adenosine substitutions in multiple positions of the D and V loops would be expected to disrupt pairing. The D and V loops also show reasonable similarity to the acceptor stems aligned above them. Ac and T loops are very similar in sequence.

Although the D loop (8–24 and 25–29) is refolded in formation of the cloverleaf tRNA, it may still show some sequence relics of the original posited 17-nt microhelix. A possible ancestral 17-nt microhelix sequence might have been close to TAGTCTAGCCTGGACTA (the posited anticodon is underlined), which can form 5/5-nt paired stems flanking a 7-nt loop with a GCC anticodon (encoding glycine). Note that GTT, GTC, and GCT anticodons also seem possible (Fig. 4). We posit that this segment of the D loop is derived from a 17-nt microhelix, which could have been part of a 31-nt minihelix. Of course, the D loop is flanked by the 5´-7-nt acceptor stem (1–7) and a 5-nt apparent remnant of an acceptor stem (25–29), indicating that the D loop may have once been derived from a 31-nt minihelix. The anticodon loop (30–46) may have had the ancestral sequence CCGGGCTXXXAACCCGG (the anticodon sequence in the initial ligation cannot now perhaps be known). In the tRNA cloverleaf, the anticodon loop is flanked by 2–5-nt apparent acceptor stem remnants (25–29 (D loop) and 47–51 (V loop)), indicating that the anticodon loop was derived from a 31-nt-minihelix. The T loop (52–68) may have had the ancestral sequence CCGGGTTCAAATCCCGG, which is remarkably similar to the proposed anticodon loop ancestral sequence. The archaeal T loop is highly conserved. The T loop appears to be derived from a CAA anticodon minihelix (encoding leucine), although a CGA anticodon minihelix (encoding serine) is possible. Because of the high conservation of the T loop in archaea, cloverleaf tRNA probably initially evolved in a single event, followed by acquisition of different anticodon loops, which we posit to have existed in the preceding 31-nt proto-tRNA minihelix world. The T loop is flanked by the 5-nt V loop (47–51) and the 3´-7-nt acceptor stem (69–75), indicating that the T loop may be derived from a 31-nt-minihelix. Ancestral coding sequences for the acceptor stems and the acceptor stem relics might be close to 1-GCGGCCG-7, 69-CGGCCGC-75, 25-GACCG-29, and 47-TGGTC-51.

So, in summary, the tRNA 75-nt core is posited to be preserved from LUCA in many archaeal tRNAs. ∼20 % of archaeal tRNAs have an intact D loop, but most bacterial and eukaryotic tRNAs have D loops that include deletions. D loop deletions are expected to inhibit the minihelix and microhelix folding patterns, which are incompatible with D loop folding in the cloverleaf. Cloverleaf tRNA appears to include a 31-nt minihelix preserved within its structure (1–7 and 52–75 (T loop 17-nt microhelix + 2–7-nt paired acceptor stems)). 3–17-nt microhelices appear in the cloverleaf structure (8–24 (D loop; refolded), 30–46 (Ac loop), and 52–68 (T loop)). Because 5-nt remnants of acceptor stems cannot be generated by ligation of segments of equal length, the proposed 5-nt acceptor stem remnants (25–29 and 47–51) were most likely generated by deletion.

**Figure 6.**
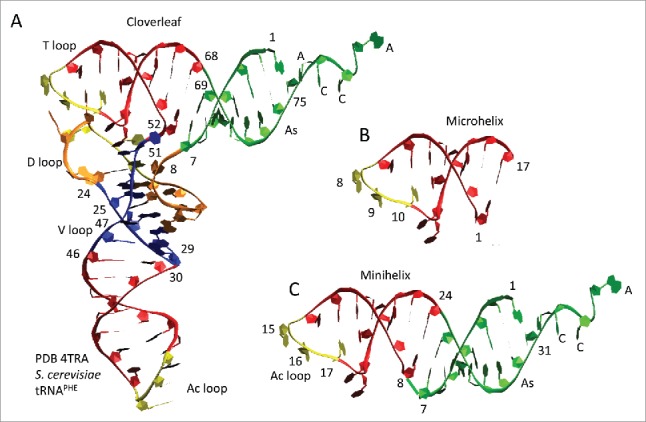
Fitting the model to the structure of the tRNA cloverleaf. (A) Cloverleaf tRNA numbered according to the 75-nt model. Coloring is as in Fig. 1B, except the D loop microhelix is colored orange instead of red to emphasize that this segment of the D loop is a refolded 17-nt microhelix. 7-nt acceptor stems (As) are green (1–7 and 69–75). The 3′-CCA end is green. 5-nt acceptor stem relics (D loop (25–29); V loop (47–51)) are blue. 17-nt microhelices are red and yellow (Ac loop (30–46); T loop (52–68)) or orange and yellow (D loop (8–24)). Anticodon derived sequences are yellow. (B) A 17-nt microhelix. (C) A 31-nt minihelix with a 3´-CCA.

A structural model of cloverleaf tRNA is shown in Figure 6, colored and numbered according to the 75-nt model (see Fig. 1B). *S. cerevisiae* tRNA^PHE^ has a 3-nt deletion in the D loop (orange segment), so the numbering was adjusted to the 75-nt model. The refolded D loop microhelix is colored orange and yellow (8–24) (Fig. 6A). The Ac loop (30–46) and T loop (52–68) microhelices are colored red and yellow. Anticodon derived sequences are yellow. Acceptor stems are green. The 3´-CCA end, where the amino acid is attached, is green. Acceptor stem relics within the D loop (25–29) and V loop (47–51) are blue. Figure 6B shows a 17-nt microhelix. Potentially, a 3´-CCA could be attached to form a 20-nt translation adaptor, lacking an acceptor stem. Figure 6C shows a 31-nt minihelix with a 3´-CCA. The 31-nt minihelix structure, which is derived from the tRNA cloverleaf, appears to represent a previous generation coding adaptor scheme. Here, we are suggesting that a 31-nt proto-tRNA minihelix world preceded the cloverleaf tRNA world, making cloverleaf tRNA at least a second-generation template-dependent translation scheme and possibly a third-generation scheme. As noted above, the 17-nt microhelix could attach a 3´-CCA to become a 20-nt adaptor lacking an acceptor stem (Fig. 6B).

## Discussion

After >3.5 billion years of evolutionary pressure on the tRNA structure, a model can be constructed that describes 75/75-nt of the tRNA conserved core. The model was developed by inspection of sequences (Figs. 1 and 3–5) and structural analysis (Figs. 2 and 6), as described in the text and in Methods. The Ac and T loops are similar in sequence and homologous in structure. We conclude that the Ac and T loops (30–46 and 52–68) are paralogs (i.e., relics of proto-tRNAs probably with different anticodons) joined by ligation. The acceptor stems (1–7 and 69–75) are recognized by amino acyl tRNA synthetases to attach a specified amino acid to the 3´-CCA tRNA end. Thus, 48/75-nt of tRNA are assigned with confidence. Because the tRNA cloverleaf includes a 31-nt minihelix structure (1–7 and 52–75), we posit that at least 2–31-nt minihelices were joined to form the tRNA cloverleaf. Because the D loop is adjacent to a 7-nt acceptor stem and an apparent 5-nt acceptor stem relic, because of the maximum D loop length in archaeal tRNAs and because some D loops have the capacity to form an in-phase stem compared to Ac and T loops, the D loop is also posited to be derived from a 31-nt minihelix. As noted above, since LUCA, the D loop is under evolutionary pressure to adopt a different fold than it would to form a 31-nt minihelix. Refolding of the D loop, therefore, is expected to force sequence changes in evolution (deletions and substitutions). Following this reasoning, the D loop is bracketed by a 7-nt acceptor stem and an apparent 5-nt acceptor stem remnant. The Ac loop is found to be bracketed by 2–5-nt remnants of acceptor stems, which may have initially been able to pair (before LUCA; as a 31-nt minihelix). The T loop is bracketed by a 5-nt acceptor stem remnant and a 7-nt acceptor stem. We posit that the 2–5-nt acceptor stem remnants were formed by two-symmetrical 9-nt deletions within joined acceptor stems (Fig. 1B). The amino-acylated CCA-3´ end, to which the amino acid or peptide chain is covalently joined, is added to tRNA enzymatically in many archaeal and eukaryotic systems.

### Protein coding in the proto-tRNA minihelix world

The posited 31-nt proto-tRNA minihelix world could have encoded the current 20 common amino acids (or a subset). From the cloverleaf tRNA structure, we have evidence for two-encoded amino acids, probably glycine (GCC) (D loop) and leucine (CAA) (T loop). Because the logos for the D and T loops are so strong in archaea (Figs. 3–5), it appears that cloverleaf tRNA evolved in a single event. LUCA is generally considered one of the first cellular and DNA genome-based organisms. How a templated protein-coding system, posited to have existed in the ancient RNA-protein world, therefore, could have been converted at LUCA to robust cloverleaf tRNA coding is important to consider. In the transition to the DNA genome world, one possible mechanism for transferring multiple anticodon loops from proto-tRNA minihelices to cloverleaf tRNAs might be through homologous DNA recombination, and RecA is known to be an ancient protein.^26^

### tRNA as a molecular fossil and proto-tRNA replication to generate complex RNAs

**Figure 7.**
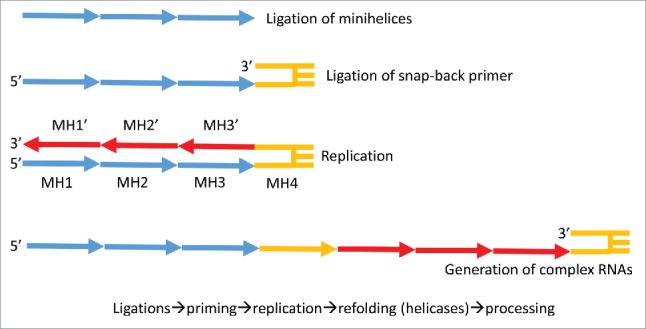
A mechanism to generate complex RNAs such as cloverleaf tRNA and proto-rRNAs via replication of 31-nt minihelices (MH). MH1 and MH1´ are complements.

**Figure 8.**
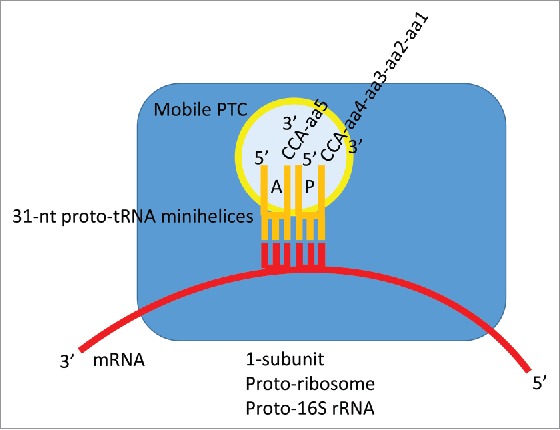
A proposed model for mRNA-encoded translation in the 31-nt proto-tRNA minihelix world. A similar model, with expected lower fidelity (no acceptor stems), could be constructed based on amino-acylated 17-nt microhelices with 3´-CCA ends.

Assuming that tRNA was generated by ligation of 3–31-nt minihelices, and assuming that the cloverleaf tRNA world was pre-dated by a 31-nt proto-tRNA minihelix world, insight is gained into the probable mechanisms for 31-nt minihelix replication (Fig. 7) and translation (Fig. 8). We propose that some RNAs in the RNA–protein world, including minihelices, were replicated by ligation and snapback priming, often primed by 3′ ligation of a 31-nt minihelix (lacking a 3′-CCA (Fig. 7)). Such a replication mechanism generates long RNAs, such as cloverleaf tRNAs (with necessary internal processing) and proto-rRNAs. Essentially, the proto-tRNA minihelix world appears to be a laboratory for generation of complex and sometimes functional RNAs.

If bunches of ligated 31-nt minihelices were replication intermediates in the proto-tRNA minihelix world, many minihelix ligations, including those that generate a cloverleaf tRNA precursor, are necessary and expected. 31-nt minihelices can ligate to form snap back replication primers. Furthermore, a mechanism for excision of 31-nt minihelices from larger RNAs (i.e., generated to allow replication (Fig. 7)) is also necessary. Remarkably, from the proposed ligation of 3-minihelices, the cloverleaf tRNA is derived (with very few sequence changes) simply by two-symmetrical 9-nt deletions within the posited two-internally ligated CG-rich acceptor stems. To cause the deletions, at least two of the indicated RNA cleavages appear to occur at the base of an expected stem-loop (positions a and d; Fig. 1B). Cleavage at the base of stems is a necessary activity in the proto-tRNA world, for instance, to generate mature 31-nt minihelices after replication, which probably was primed by ligation of a 31-nt hairpin minihelix (Fig. 7). The RNA cleavage activity at positions a and d (Fig. 1B) is similar to tRNA excision enzymes such as RNase P, RNase E, and RNase X. Cleavage within RNA stems, as in positions b and c (Fig. 1B), occurs with RNase III, RNase M23, RNase M16, and RNase M5.^27^ Because of the passage of time and the changing selection pressures caused by competition between the cloverleaf tRNA world and the proto-tRNA minihelix world, the order of events for stem trimming and ligations cannot perhaps now be known.

### Proto-ribosomes and proto-rRNA in the 31-nt proto-tRNA minihelix world

Judging from the structure of cloverleaf tRNA, the cellular tRNA world appears to have replaced a proto-tRNA minihelix coding world that included 31-nt minihelix proto-tRNAs with acceptor stems, anticodon loops, and amino acylated CCA-3´ ends. In keeping with the role of ribosomes as reaction scaffolds, perhaps, in the proto-tRNA world, minihelix mRNA-dependent translation may have occurred on a single pre-ribosomal subunit scaffold with a decoding center and utilizing a possibly mobile PTC, itself perhaps formed of proto-tRNAs (Fig. 8).^16,17^ For instance, a precursor of the 16S rRNA-containing ribosomal subunit, which positions the mRNA, could have formed the scaffold for minihelix-dependent translation. According to this view, the 16S rRNA would be expected to be more ancient and more highly conserved than 23S and 5S rRNA. On the cellular two subunit ribosome, the PTC is part (domain 5) of the 23S rRNA. Evolution of the tRNA cloverleaf, therefore, with its longer length from anticodon to CCA end, compared to a 31-nt minihelix, may have driven evolutionary pressure toward the cellular 2-subunit ribosome from a 1-subunit proto-ribosome constructed around proto-16S rRNA.

### Iteration in evolution

The results presented here provide striking evidence of the power of using the iteration of simple motifs to build complexity in ancient molecular evolution. As with (β−α)_8_ proteins, RNA polymerases, general transcription factors, promoters and glucose-binding modules, tRNA sequences, and structures can be read back with surprising confidence >3.5 billion years. Surprisingly, the tRNA cloverleaf appears to have evolved in a single event by ligation of 3–31-nt proto-tRNA minihelices encoding glycine (GCC), an unknown amino acid and leucine (CAA). Two symmetrical 9-nt deletions in ligated acceptor stems brought the tRNA cloverleaf core to 75-nt. At the core of life, evolution is remarkably conservative, and the 75-nt tRNA cloverleaf core reflects an ancient event. Because biology is a written language, records of molecular evolution are preserved from deepest antiquity (>3.5 billion years) in nucleic acid and protein primary, secondary, and tertiary sequences and structures.

## Methods

The model for tRNA evolution was developed by inspection of tRNA structures, cloverleaf diagrams, and sequences. The tRNA database (tRNAdb; http://trnadb.bioinf.uni-leipzig.de/) was used as a source of annotated tRNA sequences.^25^ To draw structural images, Visual Molecular Dynamics was used.^28^ Pymol (https://www.pymol.org/) and VMD were used to overlay tRNA Ac and T loops. Several other tRNA Ac and T loops were overlayed with similar results (i.e., PDBs 1YFG and 2AKE).^29,30^ Logos were made using WebLOGO 3.5 (http://weblogo.threeplusone.com/create.cgi).^31^ Initially, it was noticed that the Ac and T loops were likely homologs. This can be inferred by inspection of an archaeal typical tRNA sequence (Fig. 1A). Then structural overlays were done, confirming that the Ac and T loops are structurally homologous. Inspection of archaeal typical tRNA sequences revealed that many archaeal tRNAs (∼20 %) (Fig. 3) have longer D loops than almost all bacterial and eukaryotic tRNAs. Counting the length of the D loops indicated that what was described as a tRNA D loop was possibly derived from a 17-nt microhelix (8–24; refolded) and a 5-nt remnant of an acceptor stem (25–29). If the 3´-5-nt of the D loop (25–29) is a remnant of an acceptor stem, this predicts that the 5-nt V loop is also a remnant of an acceptor stem (47–51) that, before cloverleaf folding, could have been paired with the 3´-5-nt remnant of the D loop. V loops >5-nt include insertions. The model also indicates symmetry in processing of a 93-nt precursor to a 75-nt tRNA core, providing insight into RNA processing in the RNA–protein world. Inspection of D loop and V loop sequences seems consistent with our model (Figs. 3–5). For convenience, making the sequence model (Fig. 1B) was done with 3–31-nt minihelices encoding Gly (GCC), Thr (TGT) (any microhelix anticodon would do), and Leu (CAA) and identical acceptor stems (probably an incorrect assumption), using *S. solfataricus* microhelix sequences. To generate an improved sequence model requires more accurate reconstructions of proto-tRNA minihelix sequences.
